# Molecular detection of *Mycobacterium bovis* in cattle herds of the state of Pernambuco, Brazil

**DOI:** 10.1186/s12917-016-0656-1

**Published:** 2016-02-20

**Authors:** Renata Duarte da Silva Cezar, Norma Lucena-Silva, Antônio Fernando Barbosa Batista Filho, Jonas de Melo Borges, Pollyane Raysa Fernandes de Oliveira, Érica Chaves Lúcio, Maíra Arruda-Lima, Vania Lucia de Assis Santana, José Wilton Pinheiro Junior

**Affiliations:** Federal Rural University of Pernambuco (Universidade Federal Rural de Pernambuco - UFRPE), Rua Dom Manuel de Medeiros, s/n, Dois Irmãos, Recife, Pernambuco CEP 52171-900 Brazil; Department of Immunology (Departamento de Imunologia), Research Center Aggeu Magalhães (Centro de Pesquisas Aggeu Magalhães-CPqAM) Oswaldo Cruz Foundation (Fundação Oswaldo Cruz - Fiocruz), Av. Professor Moraes Rego, s/n, Cidade Universitária, Recife, Pernambuco CEP 50.740-465 Brazil; Academic Unit of Garanhuns (Unidade Acadêmica de Garanhuns), Federal Rural University of Pernambuco (Universidade Federal Rural de Pernambuco – UFRPE). Avenida Bom Pastor, s/n, Boa Vista, Garanhuns, Pernambuco CEP 55292-270 Brazil; National Agricultural Laboratory of Pernambuco (Laboratório Nacional Agropecuário de Pernambuco- Lanagro/PE), Ministry of Agriculture, Livestock and Food Supply of Brazil (Ministério da Agricultura, Pecuária e Abastecimento – MAPA). Rua Manoel de Medeiros, s/n, Dois Irmãos, Recife, Pernambuco CEP 52171-030 Brazil

**Keywords:** Tuberculosis, Cows, PCR, Milk

## Abstract

**Background:**

The present study aimed to direct detect *Mycobacterium bovis* in milk (*n* = 401) and blood (*n* = 401) samples collected from 401 dairy cows of 20 properties located in the state of Pernambuco, Brazil, by real-time quantitative PCR (qPCR) targeting the region of difference 4 (RD4). Risk factors possibly associated with bovine tuberculosis (BTB) were also evaluated.

**Results:**

Of the 802 samples analyzed, one milk (0.25 %) and eight blood (2 %) samples were positive for *M. bovis* in the qPCR and their identities were confirmed by sequencing. Animals positive for *M. bovis* were found in six (30 %) of the 20 properties visited. None of the risk factors evaluated were statistically associated with BTB.

**Conclusions:**

*M. bovis* DNA was detected in one milk sample what may pose a risk to public health because raw milk is commonly consumed in Brazil.

## Background

Bovine tuberculosis (BTB) is caused by *Mycobacterium bovis*, a member of the *Mycobacterium tuberculosis* complex that affects mammals, including humans [1].

*M. bovis* has been isolated from milk and colostrum samples what can be important to perpetuate BTB infection in a herd through the digestive route [2]. Raw milk is commonly consumed in Brazil [3] and clandestine milk is an important public health issue in the country [4, 5].

Despite the fact that Brazil has a National Program for Control and Eradication of Tuberculosis and Brucellosis (Programa Nacional de Controle e Erradicação da Tuberculose e da Brucelose - PNCETB) supervised by a public agency, its implementation is not mandatory [6]. The tuberculin skin test is the diagnostic method recommended by the PNCETB and it must be followed by bacterial isolation for result confirmation [7]. Efforts to reduce the risk of *M. bovis* infection must include sanitary measures to ensure a healthy cattle herd.

Molecular techniques such as Polymerase Chain Reaction (PCR) have been used for BTB diagnosis in several clinical samples such as blood, milk and nasal exudates [2]. Standardization of direct methods for detection of *M. bovis* in clinical samples will enable a more accurate BTB diagnosis and facilitate epidemiological studies on *M. bovis* prevalence [1, 8].

In the present study, we used qPCR for direct detection of *Mycobacterium bovis* in milk and blood samples of cattle from the state of Pernambuco, Brazil.

## Methods

### Sampling

The sample size was calculated as recommended by Thrusfield [9] using the following parameters: bovine population of 336,221 animals in the micro region of Garanhuns, state of Pernambuco, Brazil [10], 95 % confidence interval and 5 % sampling error margin using a prevalence of 50 %, since there is no official data on BTB prevalence in the studied region. According to the calculation, the minimum sample size should be 385 dairy cattle.

From January to February 2014, a total of 802 milk and blood samples were collected from 401 dairy cows of 20 properties distributed in the municipalities of Angelim, Bom Conselho, Brejão, Caetés, Calçado, Canhotinho, Correntes, Garanhuns, Iati, Jucati, Jupi, Jurema, Lagoa do Ouro, Lajedo, Palmeirina, Paranatama, Saloá, São João and Terezinha, state of Pernambuco, Brazil.

Written informed consent was obtained from the farmers to take samples from the cattle. The blood samples (*n* = 401) were collected by caudal venipuncture, stored in tubes containing citrate, properly identified and sent to the Garanhuns Laboratories Center (Central de Laboratórios de Garanhuns - CENLAG), located in the Garanhuns Academic Unit (Unidade Acadêmica de Garanhuns - UAG) of the Federal Rural University of Pernambuco (Universidade Federal Rural de Pernambuco - UFRPE), Brazil.

The milk samples (*n* = 401), which consisted of 50 ml of a pool of milk from the four quarters of each cow, were collected during milking after the udder disinfection with 70 % alcohol and the first jets of milk were discarded. Then the samples were stored in sterile bottles, cooled and sent to the CENLAG (UAG- UFRPE).

An epidemiological questionnaire containing multiple-choice questions concerning animal production characteristics, hygiene and sanitary aspects of the herd, and reproductive management was applied in each property. The questionnaire comprised 11 possible risk factors for *M. bovis* infection, as follows: herd size (less than 50 animals, 51–100 animals, 101–200 animals, more than 201 animals), rearing system (intensive, extensive, semi-intensive), origin of replacement animals (farm’s own herd, another farm, both), conducting quarantine after animal’s purchase, performance of BTB diagnostic tests upon animals’ acquisition, water source (stagnant or running), milking procedure (manual or mechanic), frequency of cleaning the farm facilities, udder disinfection, feeding colostrum to calves and history of BTB in the herd.

### DNA Extraction

*M. bovis* DNA was extracted from milk samples using the QIamp® kit (Qiagen Inc.) following the manufacturer’s instructions. Leukocyte DNA was isolated by a modified phenol-chloroform extraction method [11], 100 μl of white blood cells were used, ammonium acetate and phenol-chloroform for DNA extraction from the blood samples.

### Positive control

The *Mycobacterium bovis* ATCC 19274 strain was gently provided by the Oswaldo Cruz Foundation (Fundação Osvaldo Cruz - FIOCRUZ, Rio de Janeiro, Brazil) and was used for construction of a plasmid harboring the target sequence, which was the positive control in the molecular assays. *M. bovis* genomic DNA was extracted and the fragment corresponding to the region of difference 4 (RD4) was amplified with the specific primers reported by Sales et al*.* [12]. The target fragment was cloned using *Escherichia coli* XL1 blue strain and TA cloning kit® (Invitrogen) according to the manufacturer's instructions. The recombinant plasmid pRD4-TA was sequenced by the Sanger method using an ABI3100 Genetic Analyzer (Applied Biosystems).

### Real-time PCR

The molecular detection of *M. bovis* DNA in the milk and blood samples was performed in the Laboratory of Immunogenetics (Laboratório de Imunogenética) of FIOCRUZ, state of Pernambuco, Brazil. Quantitative real time PCR (qPCR) was performed using the same primer set used for amplification of the RD4 fragment of the positive control and a fluorescent probe that discriminates *M. bovis* from other *M. tuberculosis* complex members since it hybridizes with both the 5′ and 3′ RD4 deletion flanking sequences, which only occur directly adjacent to each other in *M. bovis*. The probe was designed using the software Primer Express® Software and targeted a region in the amplicon in between the primer pair. The probe showed 100 % homology to *M. bovis* in BLAST/ncbi. Probe sequence: 5’- /56-FAM/AGCCGTAGTCGTGCAGAAGCGCA/3BHQ_1/- 3’. The total reaction volume was 25 μL comprising 2.0 μL of DNA, 12.5 μL of TaqMan®Universal PCR Master Mix, 1.0 μL each primer (5 pmol), 0.5 μL of probe (5 pmol) and water (8 μL). The amplification conditions were 95 °C for 15 min (denaturation) followed by 40 cycles of 94 °C for 15 s and 60 °C for 60 s. In all PCR runs, standard curves were obtained using the positive control, plasmid DNA encompassing the mycobacteria RD4 sequence, which was prepared in triplicate by serial dilution of 10x plasmid DNA from 200 ng (Quantification cycle - Cq = 11.8) to 0.0002 ng (Cq = 32.2). The qPCR was performed in an ABI 7500 Real-Time PCR system set for absolute quantification The slope of the standard curve was −3.40 and R = 0.999, with 97 % efficiency.

Spiked samples were not used. The standard curve and the detection limit were determined using a serial 10X dilution from 100 to 10^−10^ ng/ul in triplicate and the positive control was detected in samples with up to 10^−6^ ng/ul. The reaction was repeated four times in different days and the same results were obtained in each day.

To verify the presence of inhibitors in the samples, a few blood samples of 1000 ng/μl DNA were randomly selected and diluted them to 800, 600, 400, 200 and 50 ng/μl of DNA. Then 20 ng were added of positive control to the diluted samples and performed a qPCR. All the dilutions had the same Cq in the qPCR; therefore, there were no inhibitors in the samples.

### Sequencing

DNA sequencing was performed in the Center of Technological Platforms (Núcleo de Plataformas Tecnológicas - NPT), of the Research Center Aggeu Magalhães (Centro de Pesquisas Aggeu Magalhães-CPqAM), FIOCRUZ, state of Pernambuco, Brazil.

The commercial kit ABI PRISM BigDye Terminator Cycle Sequencing Ready Reaction v3.1 (Applied Biosystems®) was used for DNA sequencing following the manufacturer’s recommendations. The RD4 fragments were sequenced by the Sanger method and the reaction products were analyzed in the ABI 3500xL Genetic Analyzer (Applied Biosystems).

All the sequences obtained in the present study were compared with the RD4 fragment of the reference genome (88 bp) (GenBank Access number BX248339.1) using the software Blast-N (http://www.ncbi.nlm.nih.gov) and MEGA6 [13].

### Ethical considerations

The Ethics Committee on Animal Use (Comissão de Ética no Uso de Animais – CEUA) of UFRPE provided scientific and ethical clearance for the present study (reference number 23082.004671/2013, license number 028/2013).

### Statistical analysis

The absolute and relative prevalence of *M. bovis* in the milk and blood samples were determined by descriptive analysis. Univariate analysis using chi-square test, Pearson’s test or Fisher’s exact test were used to evaluate the possible risk factors associated with BTB. All statistical analyzes were performed in the Epi Info 3.5.1 software.

## Results

Of the 802 samples analyzed, one milk (0.25 %) and eight blood (2 %) samples were positive for *M. bovis* in the qPCR and their identities were confirmed by sequencing. All positive samples were from different animals (Table 1).

**Table 1:** Results of qPCR of milk and blood samples collected from cattle of the micro region of Garanhuns, state of Pernambuco, Brazil, 2014

	Number of animals	Positive		Negative	
Municipality^a^		Milk	Blood	Milk	Blood
Bom Conselho	25	-	01	25	24
Lagoa do Ouro	40	-	01	40	39
Paranatama	18	-	01	18	17
Iati	13	-	01	13	12
Caetés	18	01	-	17	18
Palmerina	10	-	04	10	06
Total^b^	401	01	08	400	393

^a^Municipalities which herds had only negative results in the qPCR are not shown

^b^Refers to the total number of animals evaluated in the study; it is not a sum of each column

Six (30 %) of the 20 properties visited had animals positive for *M. bovis* (Table 1). None of the risk factors evaluated in the present study were statistically associated with BTB as shown in Table 2.

**Table 2:** Analysis of risk factors associated with prevalence of *M. bovis* in cattle herds of the micro region of Garanhuns, state of Pernambuco, Brazil, 2014

Risk factors	n	Positive		Negative		OR (95 % CI)	*p* value
		AF	RF%	AF	RF%		
Herd size^a^							
< 50 animals	9	2	22.2	7	77.8	-	0.467
51–100 animals	6	3	50.0	3	50.0	3.50 (0.37–32.97)	
101–200 animals	2	1	50.0	1	50.0	1.00 (0.04–24.55)	
> 200 animals	2	-	-	2	100	-	
Rearing system							
Intensive	3	1	33.3	2	66.7	-	0.788
Extensive	2	1	50.0	1	50.0	2.00 (0.05–78.25)	
Semi-Intensive	15	4	26.7	11	73.3	0.36 (0.02–7.30)	
Origin of replacement animals							
Farm’s own herd	12	4	33.3	8	66.7	0.67 (0.09–4.92)	0.544
Other farms	8	2	25.0	6	75.0		
Quarantine							
Yes	4	1	25.0	3	75.0	1.36 (0.11–16.57)	0.657
No	16	5	31.3	11	68.8		
BTB diagnostic tests upon animals’ acquisition^a^							
Yes	10	3	30.0	7	70.0	1.16 (0.16–8.0)	0.630
No	9	3	33.3	6	66.7		
Water source^a^							
Stagnant	14	4	28.6	10	71.4	1.66 (0.19–14.0)	0.520
Running	5	2	40.0	3	60.0		
Milking procedure							
Manual	12	4	33.3	8	66.7	0.66 (0.09–4.92)	0.544
Mechanic	8	2	25.0	6	75.0		
Frequency of cleaning the farm facilities^a^							
Daily	12	3	25.0	9	75.0	-	0.360
Weekly	3	2	66.7	1	33.3	6.00 (0.39–92.28)	
Monthly	2	1	50.0	1	50.0	0.50 (0.01–19.56)	
Udder disinfection							
Yes	7	1	14.3	6	85.7	3.75 (0.34–41.0	0.276
No	13	5	38.5	8	61.5		
Feeding colostrum to calves							
Yes	17	5	29.4	12	70.6	1.20 (0.08–16.44)	0.370
No	3	1	33.3	2	66.7		
History of bovine tuberculosis in the herd^a^							
Yes	2	-	-	2	100.0	-	0.456
No	17	6	35.3	11	64.6		

*AF* absolute frequency, *RF* relative frequency, *OR* odds ratio, *95 % CI* 95 % confidence interval

^a^Not all the respondents answered the question

## Discussion

This is the first report of direct detection of *M. bovis* DNA in milk and blood samples from cattle of the region of Garanhuns, state of Pernambuco, Brazil.

The prevalence of *M. bovis* DNA in milk samples ranges from 2 to 87 % according to different studies [14–20], which evaluated the mycobacteria presence by PCR. The different prevalence rates observed by them may be related to management characteristics [21], sampling methods [22] and disease-control measures adopted in each location [23].

The presence of *M. bovis* in milk may pose a risk to public health, because humans can become infected by *M. bovis* through exposure to infected animals, consumption of infected raw milk and dairy products [24, 25]. The presence of *M. bovis* in milk samples is a concern because it is estimated that 41 % of all milk consumed in Brazil is not pasteurized [12] being a source of infection of human TB. Since the clinical symptoms of human TB caused by *M. bovis* are indistinguishable from those caused by *M. tuberculosis* [23, 26], a detailed epidemiological investigation considering the patients eating habits and professional occupation must be performed by the health surveillance service to determine the TB causal agent. In Brazil, a study performed by Silva et al*.* [27] with 189 TB patients identified co-infection with *M. bovis* in three patients. In two of these patients, consumption of cheese made with raw milk was the probable cause of infection. The other patient used to work in a slaughterhouse, so the infection was related to labor risk. Other study conducted in Brazil also identified *M. bovis* in humans, although with lower prevalence. It is believed that *M. bovis* prevalence in Brazil is underestimated [28].

In the present study, the milk sample positive for *M. bovis* did not belong to any of the eight animals that had positive blood samples what can be explained by various reasons: collection of only one milk sample per dairy cow, interaction between the bacillus and the bovine immune system cells, which may have decreased the amount of bacillus in milk [2, 20, 29], and presence of milk proteins and fat that may have impaired the extraction of *M. bovis* DNA from the milk samples [14, 30]. Despite these limitations, studies using experimentally contaminated milk have demonstrated that PCR can detect the mycobacteria in milk samples with much lower concentrations of *M. bovis* DNA than the concentration usually present in natural infections [2, 20, 29, 31]. The mycobacteria has been more frequently identified in blood than in milk samples [20, 32, 33].

Another factor that can hinder the detection of *M. bovis* in milk is its intermittent release during a short period post-infection [20, 29]. Pardo et al*.* [34] evaluated the mycobacteria secretion pattern in 780 milk samples collected from 52 animals for 15 consecutive days and *M. bovis* showed an intermittent and irregular release pattern in 26.5 % of the samples [34].

In the present study, of the six farms that had animals positive for *M. bovis*, only one have a history of performing tuberculin tests upon acquisition of new animals. According to the farmer, all the animals were negative for *M. bovis* in the tuberculin tests. This data shows the importance of using tests that are more sensitive in enzootic areas, as the state of Pernambuco, Brazil, where cases of BTB have already been identified using tuberculin skin test [35, 36].

Although there was no statistical association between herd size and *M. bovis* positivity, the mycobacteria was more prevalent in larger herds (101–200 animals). Herd size can influence BTB epidemiology because a high population density favors a more frequent contact between animals, facilitating the mycobacteria dissemination [37, 38].

According to Skuce et al. [39], *M. bovis* can survive in water what favors its dissemination. *M. bovis* DNA was identified in water samples experimentally contaminated even 11 months after contamination [40]. In Uganda, Africa, where cattle commonly drinks running water from rivers or streams, a study evaluated the risk factors associated with BTB and concluded that the water source was statistically associated with the disease [41]. However, in the present study, no correlation was found between water source and presence of *M. bovis* in milk and blood samples (Table 1)*.* The fact that water sources could be implicated with BTB transmission may be a concern to health authorities, because control measures would also have to consider this contamination source besides slaughter of positive animals.

Despite the higher number of animals positive for *M. bovis* in herds with low frequency of cleaning the farm facilities and lack of udder disinfection before milking, no positive correlation was found between pathogen presence and these risk factors. Roxo [42] reported that cleaning, disinfection and hygiene are risk factors for TB. Waste management and treatment of organic matter can influence *M. bovis* prevalence in areas with previous cases of TB [43].

## Conclusion

*M. bovis* DNA was detected in one milk sample what may pose a risk to public health. We suggest that environmental control measures should be implemented in farms at high risk of TB transmission because environmental factors contribute to bacteria perpetuation and dissemination.
